# Magnetic Properties of a High-Pressure Torsion Deformed Co-Zr Alloy

**DOI:** 10.3390/nano13162280

**Published:** 2023-08-08

**Authors:** Martin Stückler, Stefan Wurster, Markus Alfreider, Michael Zawodzki, Heinz Krenn, Andrea Bachmaier

**Affiliations:** 1 Erich Schmid Institute of Materials Science of the Austrian Academy of Sciences, 8700 Leoben, Austriastefan.wurster@oeaw.ac.at (S.W.); michael.zawodzki@oeaw.ac.at (M.Z.); 2 Department Materials Science, Montanuniversität Leoben, 8700 Leoben, Austria; markus.alfreider@unileoben.ac.at; 3 Institute of Physics, University of Graz, 8010 Graz, Austria; heinz.krenn@uni-graz.ac.at

**Keywords:** severe plastic deformation, amorphous alloys, magnetic properties, nanocrystallization

## Abstract

Co-Zr amorphous alloys exhibit soft magnetic properties, whereas the Co-rich crystalline magnetic phases in this alloy system displayed a hard magnetic behavior. In this study, an initial two-phase Co-Zr composite with an overall composition of 75 at.% Co and 25 at.% Zr was processed by high-pressure torsion (HPT), and the effects of severe plastic deformation and subsequent thermal treatment on the composite’s structural evolution and its magnetic properties were investigated. HPT processing allowed us to achieve an amorphous microstructure with low coercivity in its as-deformed state. To further tune the alloy’s magnetic properties and study its crystallization behavior, various annealed states were investigated. The microstructural properties were correlated with the magnetic properties, and a decreasing coercivity with increasing annealing temperatures was observed despite the onset of crystallization in the amorphous alloy. At higher annealing temperatures, coercivity increased again. The results appear promising for obtaining tuneable rare-earth free magnetic materials by severe plastic deformation.

## 1. Introduction

The use of severe plastic deformation by high-pressure torsion (HPT) for the production of bulk magnetic materials has been demonstrated in several studies, and reviews summarizing the results have recently been published [1,2]. For example, HPT-deformed SmCo-Fe composites have been investigated regarding their hard magnetic properties [3,4]. In the field of soft magnetic materials, HPT-deformed supersaturated Co-Cu solid solutions have provided tunability for magnetic moments and coercivity by varying the Co-to-Cu ratios [5,6]. Soft magnetic properties have been observed for high Co-content materials [5], and further improvements were achieved by substituting small amounts of Co with Fe [7]. Some commercial soft magnetic materials consist of an amorphous matrix in which a crystalline ferromagnetic phase is embedded [8]. Thus, further improvements in terms of soft magnetic properties are expected when using the formation of a similar microstructure by HPT. To achieve this goal, combinations of elements which enable the formation of an amorphous structure must be used. During mechanical alloying, the formation of amorphous microstructures in a large variety of systems, for example, by using combinations of transition metals with Zr and Ti, have already been observed [9,10,11,12,13]. Amorphization during HPT deformation has also been reported for certain material combinations and compositions such as TiNi and CuZr [14,15,16].

For the production of amorphous materials, there is, in addition to the typically used non-equilibrium processes, the possibility of using a solid-state crystalline-to-amorphous transformation. Machon and Mélinon described the occurrence of this solid–solid transformation due to an uplift of the energy minimums of the crystalline phases by substantial increases in defects (grain boundaries, dislocations, etc.), and at a certain critical defect density, a crystalline–amorphous transformation occurred [17].

In this context, HPT can be considered as a type of bulk mechanical alloying process. It is a severe plastic deformation process where a specimen is confined between two anvils featuring a cylindrical cavity. The specimen is subjected to great hydrostatic pressure, and when rotating one anvil against the other, the material is subjected to large shear deformations. The equivalent plastic strain applied to the material is radial-dependent and can be calculated according as follows:

(1) $$\varepsilon =\frac{2\pi nr}{t\sqrt{3}}$$

where *n* is the number of rotations, *r* is the radius, and *t* is the thickness of the specimen [18]. For an extensive review of deformation-induced amorphization, see [19].

In this study, HPT-induced amorphization in a Co-Zr system and magnetic properties in as-deformed and annealed states are investigated. In the 1980s and 1990s, much research was conducted on Co-Zr amorphous soft magnetic materials due to their low coercive force, high permeability, and high saturation magnetization. These properties made them suitable for use in magnetic recording read heads [20]. Shimada and Kojima demonstrated that annealing Co-Zr amorphous thin films had strongly positive effects on their coercivity [21]. Depending on the composition (5–17 at.% Zr), soft magnetic properties (Hc < 1 Oe) were achieved for a temperature range of 300 °C to 500 °C [21]. A high saturation magnetization in combination with a relatively low magnetostriction was also reported. Naoe et al. [22] showed that saturation magnetization decreases with increasing Zr contents. A high saturation magnetization can be achieved when the Zr content is as low as 2 at.%. The amorphization of Co-Zr during ball-milling has been reported to be successful for compositions of 27–92 at.% Co [11,23]. Furthermore, it is important to note that different Co-Zr phases with lower Zr contents can exhibit hard magnetic behaviors [24].

The overall composition of the HPT-deformed samples in this study was 75 at.% Co and 25 at.% Zr. There were several reasons for this choice of composition. Firstly, it lies right within the window of amorphization via ball-milling. Secondly, it was close to the transition metal Zr composition, where amorphization was already induced via HPT deformation for applied equivalent shear strains above ε = 400 [15]. Finally, the composition is close to the cubic Laves phase compound for ZrCo_2_ [25]. It has been shown that ZrCo_2_ can accommodate excess Co up to the composition for ZrCo_3_ and that the magnetic properties strongly depend on the amount of excess Co. ZrCo_2_ is strongly paramagnetic at room temperature. Ferromagnetism has been observed in ZrCo*_x_* for 2.8 ≤ *x* ≤ 3.0. [26,27].

To achieve soft magnetic properties in a severely deformed material, the idea is to induce an amorphous microstructure using HPT in which a ferromagnetic phase is embedded by subsequent annealing at a low temperature. This ferromagnetic phase might be either the aforementioned Co-rich ZrCo_x_ phase or other hard magnetic phases comprised of slightly lower Zr contents. Further tuning of the magnetic properties might be possible by prolonged annealing or by applying higher annealing temperatures for short times.

For processing the Co-Zr composite material, the HPT multi-sector disc method was used [28]. This method combines the advantages of HPT-deformed bulk- samples (i.e., no powder processing, less oxidation, and fewer impurities due to the lower amounts of free surfaces, along with uncomplicated storage and handling in an inert gas atmosphere, which is even more crucial for Zr powders) with the advantage of powder HPT (i.e., no need to pre-process the material before ball-milling or arc-melting for “mixing” the desired material). Simultaneously, the ability to process any desired material composition was maintained.

## 2. Material and Methods

The following initial materials were used for sample processing: bulk Co, purity of 99.95%, MaTeck, and bulk Zr (Zr 702, from ATI, min. Zr content of 99.2 wt% with Hf being the main alloying element (max. 4.5%)). For the multi-sector disc method, 6 bulk segments in total were produced by electro-discharge machining. The central angles for each of the 3 Zr pieces and 3 Co pieces were 50° and 70°, respectively. Taking into account the nominal mass density, the overall composition corresponded to that of Co_3_Zr. The actual HPT sample was made from these individual sectors stacked alternatingly together to build a disk with a diameter of 30 mm and a thickness of 6.35 mm. The HPT deformation was performed at room temperature using a nominal pressure of 5 GPa. Due to the discontinuous outflow of the material from the anvil cavities, the anvils came into contact during the deformation process and the HPT deformation had to be stopped. To increase the amount of applied strain, the cavity depth of the anvils was lowered twice. This resulted in a reduction in the initial sample height from 6.35 mm to 4.00 mm, 3.50 mm, and 2.00 mm after the first, second, and third HPT processing steps, respectively. The used anvils’ cavity depths for the three steps were equivalent to one-half of the resulting sample height, i.e., 2 mm, 1.75 mm, and 1 mm, respectively. The applied number of rotations for the individual steps were 65, 5, and 5.5 rotations, respectively. The applied equivalent strains for each processing step were added according to Equation (1), resulting in a maximum applied strain of ε~1100 for r = 15 mm after the last HPT processing step.

Vickers hardness measurements were made in the axial direction using a load of 1000 gf on a microindentation hardness tester from Buehler (Micromet 5104). Indents were made along the radius at a distance of 0.25 mm in the axial direction. The initial X-ray diffraction (XRD) measurements of the as-deformed state were carried out with a Phaser Bruker D2 diffractometer. The chemical composition was confirmed using an energy dispersive X-ray spectroscope (EDX, e^-^flash, Bruker, Billerica, MA, USA) attached to a scanning electron microscope (Magna, Tescan, Brno, Czech Republic).

To conduct the annealing treatments, the HPT-deformed samples were annealed at different temperatures (300 °C, 400 °C, 500 °C, and 600 °C, each for 1 h) in a conventional furnace. An additional sample was annealed at 600 °C for 100 h in a vacuum furnace.

DC-hysteresis measurements were performed using a superconducting quantum interference device (SQUID, MPMS-XL-7, Quantum Design, Darmstadt, Germany) at 300 K in magnetic fields of up to 7 T. The results of the hysteresis measurements were corrected using a Pd standard, yielding more accurate results for the coercivity (H_C_). Saturation magnetization was determined by extrapolating the mass magnetization at high fields as a function of 1/H to zero. For the chosen specimens, zero-field-cooling/field-cooling (ZFC-FC) measurements were recorded between 5 K and 300 K at 5 mT.

The deformed and annealed samples were further investigated using synchrotron high energy XRD in transmission with a beam energy of 87.1 keV (112.5 keV for the 100 h-annealed sample) at Deutsches Elektronen-Synchrotron DESY, Hamburg, Germany. To study the microstructures of the samples annealed for 1 h at 300 °C and 600 °C, transmission electron microscopy (TEM) investigations (2200-FS, JEOL, Akishima, Japan) were conducted.

## 3. Results and Discussion

**Figure 1:**
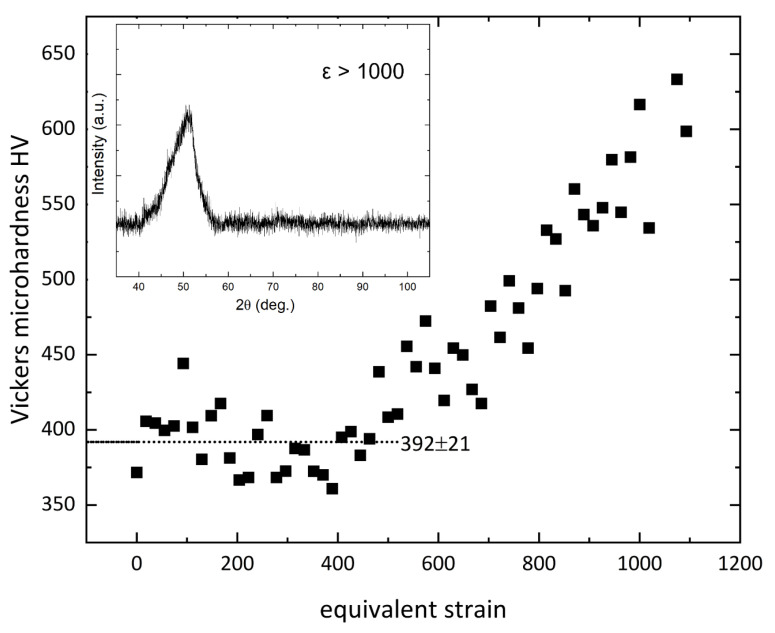
Vickers hardness as a function of equivalent strain. The inset shows the XRD pattern from the HPT-deformed Co-Zr sample taken from the radius r > 14 mm (i.e., a strain of ε > 1000).

Using EDX, the chemical composition of the sample was determined to be 76.5 ± 1.6 at.% Co and 23.5 ± 1.6 at.% Zr. In Figure 1, the hardness is plotted for the as-deformed sample as a function of the equivalent strain. Due to the three-step HPT process, the equivalent strain could only be considered as an estimate. For a radius of <6 mm, which corresponded to the equivalent strain below ε~425, a rather constant microhardness with a mean value of 392 ± 21 HV was measured. For higher applied strains (i.e., increasing sample radii), a steady increase in hardness was visible. At the outer edge of the HPT sample, the hardness reached values of approximately 600 HV, which were significantly higher than the hardness values in the inner plateau region. As described by Sun et al. for Cu-Zr [15], there is a certain applied strain necessary for the formation of amorphous material during HPT processing. Therein, it was formulated that this critical strain was reached at an equivalent strain of approximately 400 for Cu_29_Zr_71_. In Figure 1, an increase in hardness at approximately the same strain is visible and was attributed to the gradual formation of an amorphous phase during the HPT processing. XRD measurements at a position of r > 14 mm showed—within the detection limits of the XRD equipment—a fully amorphous material comprised of Co_3_Zr (inset in Figure 1). Thus, it could be concluded that for Co_75_Zr_25_, HPT-induced amorphization starts at an equivalent strain of approximately 400, which leads to a remarkable increase in hardness and to a final hardness of approximately 600 HV. In amorphous thin films with slightly lower Zr contents, a Vickers hardness of 600 has been reported as well [29].

For the as-deformed Co-Zr sample, which consisted of amorphous Co-Zr, a coercivity of 6.3 kA/m and a mass magnetization of 49.1 Am^2^kg^−1^ were measured. Amorphous Co-Zr has been reported to be a moderately strong ferromagnet that is magnetically soft with a high saturation magnetization [21,22,29,30]. Shimada and Kojima [21] found for Co_87_Zr_13_ samples a tremendous decrease in coercive force after annealing the sputtered film at 350 °C for 30 min. For slightly different Co contents, they found excellent soft magnetic properties for thermal treatments in a temperature window of 300–400 °C. Furthermore, Fe-based nanocrystalline alloys obtained through isothermal annealing at slightly higher temperatures than their amorphous counterparts also exhibited excellent soft magnetic properties [31]. Thus, the annealing treatments after the HPT processing of the Co-Zr were conducted in the temperature window 300 °C to 600 °C to generate microstructures which had improved soft magnetic properties. Furthermore, the higher annealing temperatures of 500 °C and 600 °C were chosen for the sake of investigating the structural evolution of the amorphous material. Thus, the HPT-deformed samples were first annealed at different temperatures (300 °C, 400 °C, 500 °C, and 600 °C) for 1 h each.

**Figure 2:**
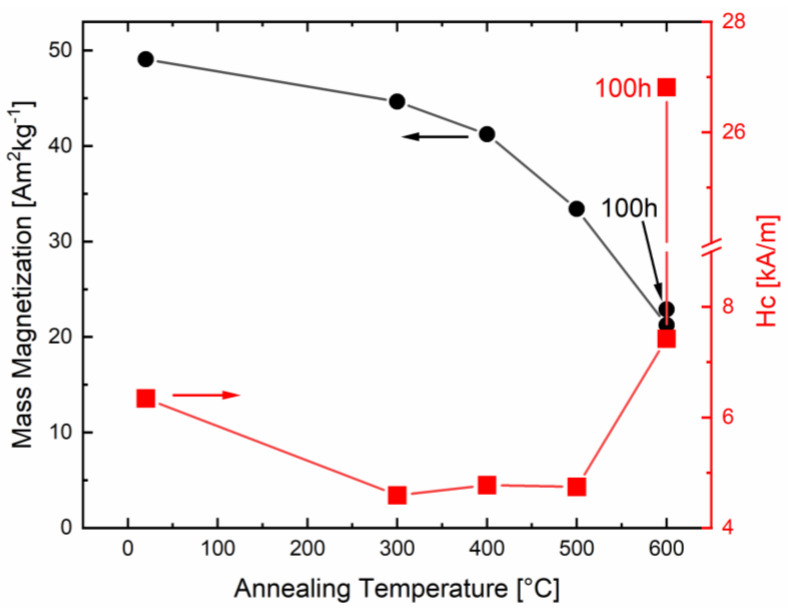
Mass magnetization and coercivity of the as-deformed state as a function of annealing temperature (300 °C, 400 °C, 500 °C, and 600 °C for 1 h each and 600 °C for 100 h; ε > 1000 for all samples). For comparison, the saturation magnetization of pure hcp Co is 162 Am^2^kg^−1^ [34].

The results of SQUID DC hysteresis measurements of the as-deformed and annealed samples are shown in Figure 2. Saturation magnetization monotonically decreases with increasing annealing temperatures, indicating the formation of non-magnetic phases. The coercivity dropped from 6.3 kA/m to 4.6–4.8 kA/m for the intermediate annealing temperatures, while it increased again to more than 7.4 kA/m for the highest annealing temperature of 600 °C. The drop at the intermediate temperatures could be attributable to a relaxation of the amorphous material at temperatures less than 300 °C, and, in particular, it could have been due to the reduction in the residual stresses upon slight annealing. It is known that HPT-processed samples exhibit large residual stresses in their as-deformed state [32,33]. At a glance, the general trend was in accordance with the results for amorphous and annealed FeCuNbSiB [31], where the initial crystallization out of the amorphous matrix led to decreased coercivity. At higher annealing temperatures, the coercivity increased again with the increasing grain size of the FeCuNbSiB alloy. However, in this study, the complex CoZr phase diagram [25] and the contributions of the different evolving phases were also taken into account.

**Figure 3:**
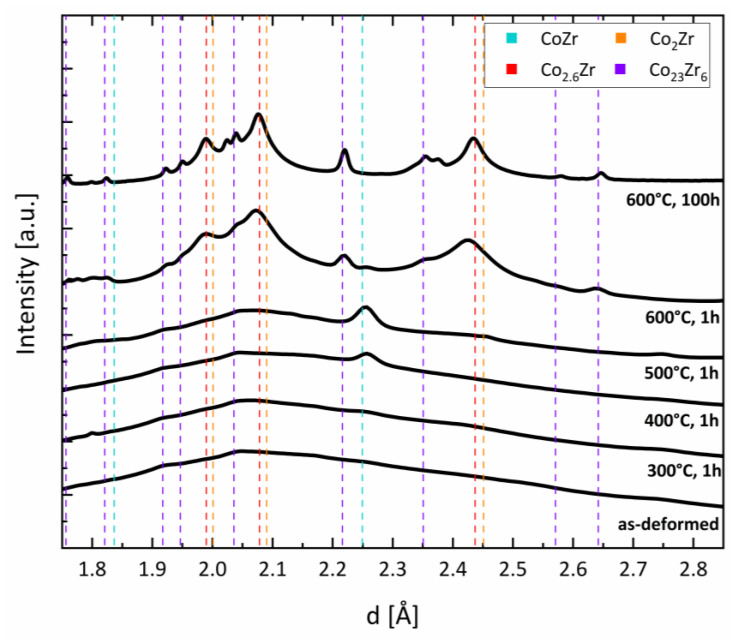
High energy XRD diffractograms for the as-deformed and annealed samples (ε > 1000). The peak positions for the CoZr, Co_2_Zr, and Co_23_Zr_6_ phases are indicated. In addition, the peak positions of the modified Co_2_Zr phases with higher Co contents (Co_2.6_Zr) are indicated (red vertical lines).

**Figure 4:**
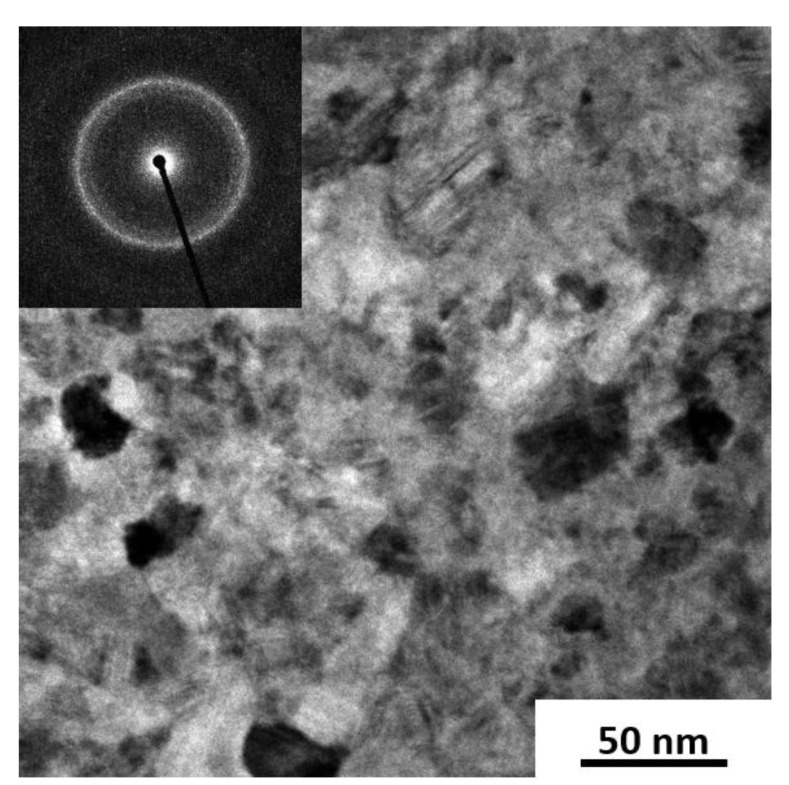
Bright field and selected area electron diffraction image of the sample annealed at 600 °C for 1 h.

For detailed phase determination, the as-deformed and annealed samples were investigated using high energy XRD (Figure 3). The high energy XRD data were evaluated using profile analysis of selected area diffraction (PASAD) software. The data in Figure 3 were obtained by integrating all the azimuthal angles [35]. The results confirmed the broad peak from the amorphous phase in the as-deformed state (ε > 1000). At the lowest annealing temperature (300 °C), the amorphous phase prevailed, which was also confirmed by the TEM investigations (not shown). Broad peaks from the amorphous phase remained visible even up to the highest annealing temperature of 600 °C. Additionally, small broad peaks at positions fitting to the cubic CoZr phase (a = 3.181Å [36]) appeared after annealing at 400 °C and 500 °C. In the cubic paramagnetic CoZr structure, the Co atoms were surrounded by non-magnetic Zr, causing it to lose its ferromagnetic character [37]. For the sample annealed at 600 °C, additional peaks at different positions appeared, whereas the peaks of the cubic CoZr phase vanished. There was better agreement in the peak positions with the Co_2_Zr phase (MgCu_2_-type), although the peak positions were slightly shifted to a smaller lattice spacing. From least-square fitting, a smaller lattice spacing of 6.893 Å was derived. Fujii [27] also found a decrease in lattice spacing with increasing the Co contents of Co_X_Zr from ~6.95 A for X = 2 to ~6.86 A for X = 3. In Figure 3, these modified peak positions (Co_2.6_Zr) are indicated as well. Fujii et al. [27] described Co_X_Zr being ferromagnetic for 2.8 < x < 3. Combining their data with those of Aoki et al. [26], they suspected an increasing ferromagnetic moment right at the composition Co_2.6_Zr; thus, the Co_2.6_Zr phase in the annealed sample may have been right at the transition from a paramagnetic state to a weakly ferromagnetic state. For the Co_3_Zr and Co_2.8_Zr, the two ferromagnetic crystalline phases investigated by Fujii et al. [27], Curie temperatures of below 200 K were found. Further evidence for the existence of a non-magnetic Co_2.6_Zr phase after the HPT deformation and annealing was the additional FC measurement (see Supplemental Figure S1) starting from 400 K. It was found that the magnetic moment increased linearly with the decreasing temperature, giving no indication of a Curie temperature below 400 K. In addition, the peaks of the magnetic Co_23_Zr_6_ phase were found after annealing at 600 °C for 1 h. The amorphous phase nearly vanished after annealing at 600 °C for 1 h, which was further confirmed by the TEM investigations that showed a nanocrystalline microstructure (Figure 4).

To further tune the magnetic properties and achieve a complete crystalline microstructure, prolonged annealing at the highest annealing temperature was performed. In Figure 3, the diffractogram of the sample annealed for 100 h at 600 °C in a vacuum is also shown. In this case, the same phases (Co_2.6_Zr and Co_23_Zr_6_) after annealing at 600 °C for 1 h were detected, but the amorphous phase vanished completely. However, few peaks in the diffractogram remained unidentified. The peak positions of these peaks did not fit to hcp and fcc Co or to pure Zr. Further, they did not match various oxides or other magnetic Co-Zr phases with lower Zr contents (ZrCo_5.1_ and Zr_2_Co_11_) which might form during an annealing treatment. For the sample annealed at 600 °C for 100 h, which consisted of crystalline Co_2.6_Zr and Co_23_Zr_6_ phases, the coercivity increased to 26.8 kA/m and the saturation magnetization changed to 22.9 Am^2^kg^−1^ (Figure 2).

**Figure 5:**
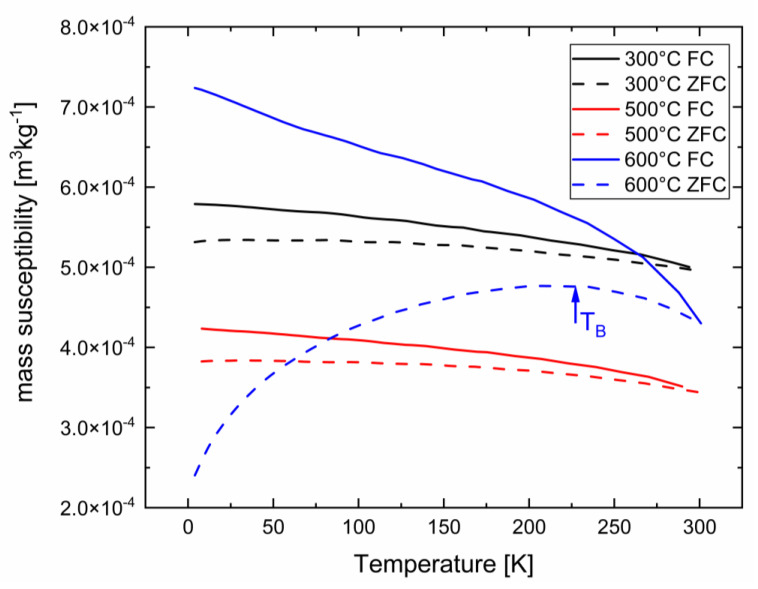
The ZFC and FC curves of the samples annealed for one hour each. In all measurements, a field of H = 5 mT was applied. The solid lines represent the FC curves and the dashed lines represent the ZFC curves. The broad hump at T_B_ is reminiscent of a “blocking” of non-relaxed ferromagnetic entities (Co_23_Zr_6_) in a non-magnetic (or weakly paramagnetic) Co_2.6_Zr matrix.

Additionally, the temperature dependence of low-field susceptibility was investigated for selected annealed samples (300 °C, 500 °C, and 600 °C for 1 h). The results are shown in Figure 5. For the ZFC measurements, a demagnetized sample was first cooled in a zero-applied field, whereas at the lowest temperature, an external field of 5 mT was applied and the magnetic moment was recorded during heating. In the FC temperature scans, the magnetic moment was measured during cooling in the same external field. The 300 °C and 500 °C annealed samples showed no splitting between the ZFC/FC scans, displaying a reversible ferromagnetic behavior. The sample annealed at 600 °C exhibited splitting and a broad peak in the ZFC-FC curve, which are typical behaviors for thermal activation and broad ferromagnetic particle distributions in non-magnetic matrices [38,39]. In summary, the increase in coercivity for the 600 °C-annealed material was due to the ferromagnetic long-range order between the Co_23_Zr_6_ phase in the non-magnetic Co_2.6_Zr phase.

In summary, the amorphous microstructure possessed a semi-hard magnetic behavior. After annealing, the soft magnetic properties were improved while mostly maintaining the amorphous state. The first crystalline diffraction peaks observed at 500 °C corresponded to the non-magnetic Co-Zr phase, whereas the peaks observed on completion of the crystallization process belonged to Co_23_Zr_6_ and formed small particles with a broad size distribution, giving rise to the measurable mass magnetization and a hyper-stoichiometric Co_2_Zr phase, with the Co_2.6_Zr likely remaining paramagnetic. For our longest annealing time of 100 h at 600 °C, the peaks became more pronounced and we again found, according to the phase diagram [25], the Co_23_Zr_6_ phase and the Co-enriched Co_2_Zr phase.

The amorphous phase in deposited CoZr alloys can be stabilized by a small amount of Zr. Typically, 5–6 at.% Zr is sufficient for achieving a uniformly amorphous structure with a room-temperature deposition [21,22]. Future work will thus consider synthesizing amorphous samples with higher Co contents by HPT deformation, followed by optimized annealing treatments. This is done, on the one hand, to achieve better soft magnetic properties due to the increased Co content, and, on the other hand, to obtain other crystallization products such as the promising rare-earth free Zr_2_Co_11_ phase [40,41] by suppressing the formation of Co_23_Zr_6_.

## 4. Conclusions

Amorphous CoZr alloys are known for their excellent soft magnetic behaviors, but hard magnetic phases are also found in this alloy system. Therefore, this paper considered the potential of severe plastic deformation for synthesizing bulk amounts of amorphous Co-Zr magnetic materials using a simple processing scheme. Using the HPT multi-sector disc method, material with the overall composition of Co_3_Zr was processed. A crystalline–amorphous transition was found at applied shear strains of ε > 400. The amorphous state was confirmed by high-energy XRD measurements. Annealing treatments at temperatures of up to 600 °C for 1 h were performed to relax the as-deformed state and induce the growth of nanocrystalline magnetic phases. SQUID magnetometry revealed a semi-hard magnetic behavior for the amorphous state, with annealing at moderate temperatures having beneficial effects on coercivity, where a drop of ~25% compared to the as-deformed state was measured. However, the saturation magnetization decreased. Annealing at temperatures of 500 °C and above led to crystallization, and a nanocrystalline microstructure for the highest annealing temperature was found. Coercivity increased with the formation of the magnetic Co_23_Zr_6_ out of the intermediately formed CoZr phase. The microstructural change, however, also had an impact on the saturation magnetization. The saturation magnetization for the samples that received the 1 h thermal treatment decreased with the increasing temperatures, which could be attributed to the ongoing formation of the paramagnetic Co_2_Zr phase, whose chemical composition was found to be Co_2.6_Zr. This work provides a new approach for the further development of HPT-processed Co-Zr magnetic materials with higher Co contents and optimized thermal treatments, and they are expected to show even better magnetic properties.

## Data Availability

Data will be made available on request.

## Supplementary Materials

The following supporting information can be downloaded at: https://www.mdpi.com/article/10.3390/nano13162280/s1, Figure S1: The FC curve (mass magnetization as a function of temperature) measured using the sample which was annealed at 600 °C for 1 h.
