# Supplementary material for: Magnetic Properties of a High-Pressure Torsion Deformed Co-Zr Alloy
Source: Nanomaterials (Basel). 2023 Aug 8;13(16):2280. doi: 10.3390/nano13162280 (PMC10458657; doi:10.3390/nano13162280)
Supplement: Supplementary file 1 [file nanomaterials-13-02280-s001.zip › nanomaterials-2524263-supplementary.pdf]

Supplementary Material

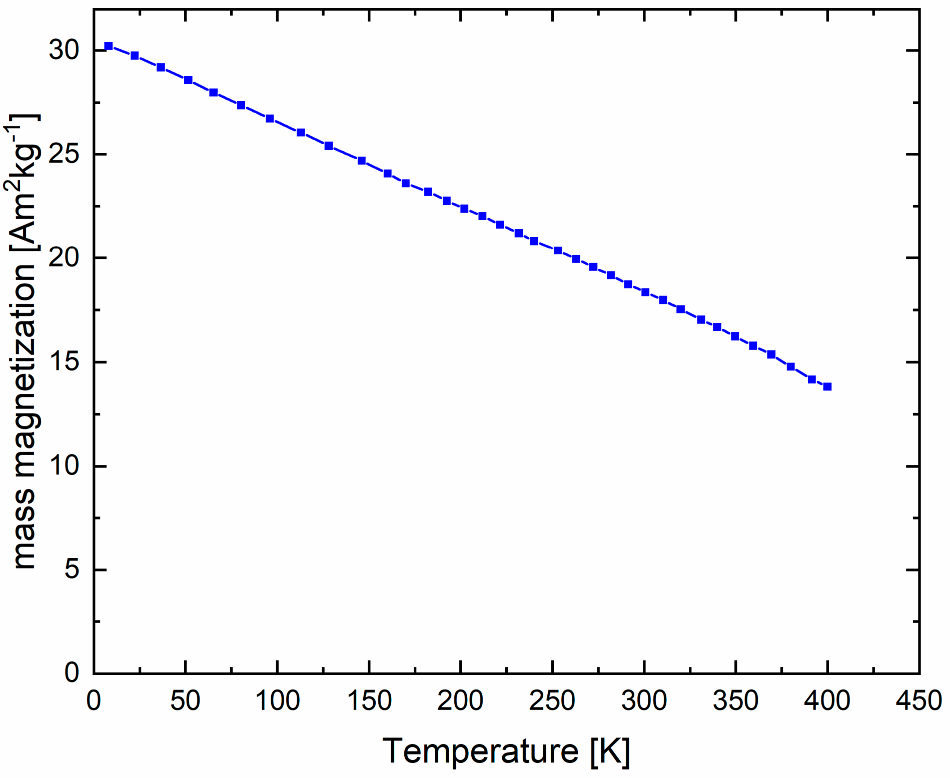

Figure S1. The FC curve (mass magnetization as a function of temperature) measured using the sample which was annealed at 600 °C for 1 h.
